# Heterologous Expression of Difficult to Produce Proteins in Bacterial Systems

**DOI:** 10.3390/ijms25020822

**Published:** 2024-01-09

**Authors:** Neus Ferrer-Miralles, Elena Garcia-Fruitós

**Affiliations:** 1 Institute for Biotechnology and Biomedicine, Autonomous University of Barcelona, 08193 Bellaterra, Spain; neus.ferrer@uab.cat; 2 Department of Genetics and Microbiology, Autonomous University of Barcelona, 08193 Bellaterra, Spain; 3 Bioengineering, Biomaterials and Nanomedicine Networking Biomedical Research Centre (CIBER-BBN), C/Monforte de Lemos 3-5, 28029 Madrid, Spain; 4 Ruminant Production Group, Institut de Recerca i Tecnologia Agroalimentàries (IRTA), 08140 Caldes de Montbui, Spain

Proteins play a crucial role in maintaining homeostasis, providing structure, and enabling various functions in biological systems. The study of proteins was a complex task before the advent of genetic engineering because they had to be obtained from natural sources with low concentrations, making purification processes challenging and yielding limited amounts. However, the development of recombinant DNA technology has allowed science to make a qualitative leap in this field of research. The production of recombinant insulin in *Escherichia coli* in the 1970s was a significant milestone, not only revolutionizing the treatment of diabetes but also initiating the use of recombinant proteins for a wide number of applications [1,2]. This breakthrough sparked a surge in biological and structural studies on proteins. Over the past nearly 50 years, the market for recombinant proteins in industries, biomedicine, and research has continued to expand, underscoring the importance of this biotechnological field [3]. Therapeutic proteins are now used in human health for the treatment of diabetes, cancer, infectious disorders, hemophilia, and anemia, and serve as indispensable tools in the diagnosis of numerous diseases [4]. In addition, recombinant proteins are used in animal production and health [5]. Moreover, the production of recombinant proteins has contributed to an exponential growth in scientific knowledge concerning the three-dimensional structure of proteins and has been instrumental in elucidating their functions [6,7]. Therefore, achieving controlled and high-performance production systems for recombinant proteins remains a top priority.

Despite advances in understanding the physiology of producing cells and the physicochemical characteristics of proteins, obtaining certain recombinant proteins still poses technological challenges due to high protein complexity and the intricacies of biological production systems. In some environments, recombinant proteins may undergo proteolysis, aggregation, or be produced in meager quantities [8]. Homologous expression systems offer an effective alternative for producing eukaryotic proteins that often undergo complex post-translational modifications. These systems create conditions favoring the accumulation of functional proteins. Nonetheless, the high cost of production processes in eukaryotic systems and the potential presence of human pathogens have led to the development of alternative yet efficient prokaryotic expression systems [9]. Among these systems, the *Escherichia coli*-based expression system has been extensively employed, utilizing a broad range of expression vectors and recombinant strains that enable the production of large quantities of heterologous proteins [10]. The technical procedures associated with these prokaryotic systems are simpler, faster, inexpensive, and easily scalable compared to most eukaryotic systems. However, certain types of proteins still pose challenges in terms of achieving sufficient quantity and quality when using a bacterial expression system. Ongoing scientific research continually reports advancements in the production processes of these difficult-to-produce proteins [11]. Access to tools and strategies that facilitate the production of such proteins is crucial for meeting the scientific and healthcare demands of our society. Therefore, it is a significant milestone for the entire scientific community specializing in this field.

This new Special Issue entitled “Heterologous Expression of Difficult-to-Produce Proteins in Bacterial Systems” of the *International Journal of Molecular Sciences* includes a total of nine contributions—four original articles and five reviews—that compile the latest biotechnological advancements, which enable the full utilization of the bacterial expression system’s potential in obtaining difficult-to-produce heterologous proteins.

One important challenge in recombinant protein production processes using bacteria is post-translational modification. Among these modifications, disulfide bond formation is especially relevant since the folding of significant number of proteins depends on it. Thus, the development of strategies to produce functional proteins containing cysteines, which form disulfide bonds, is highly relevant in a reducing environment such as the *E. coli* cytoplasm. Commercial strains with mutations in the two genes involved in the reduction pathway (*gor* and *trxB*) were developed [3]. In addition, a CyDisCo (cytoplasmic disulfide bond formation in *E. coli*) system was developed to produce proteins rich in disulfide bonds. The CyDisCo system is based on the co- or pre-expression of enzymes involved in disulfide bond formation and isomerization, which has proven to be a good strategy for proteins that have between one and five disulfide bonds [12]. Taking a step further, an article published in the Special Issue has shown the potential of this system to produce mammalian extracellular matrix (ECM) proteins containing between 8 and 44 disulfide bonds, proving that this system has no upper limits for disulfide formation, making it possible to produce complex disulfide-bonded proteins in *E. coli* BL21(DE3). Moreover, Tungekar and Ruddock have recently noted that the CyDisCo system can also be used to recombinantly produce IgG1-based Fc fusion proteins [13].

On many occasions, overproduced proteins are toxic for the producer host, inhibiting its growth and having a final low protein yield. For that reason, inducible systems are widely used [14,15,16]. However, most of these systems are only regulated at the transcriptional level to avoid any leakage expression, which frequently occurs. Thus, a complete suppression of leakage expression is needed, especially for toxic proteins. It has been observed that a complete suppression of the expression can only be controlled by dual transcriptional–translational control. In the review published in this Special Issue, Kato summarizes the principles of this dual control and lists examples of this approach, including the use of site-specific unnatural amino acid incorporation, riboswitches, ribozymes, and antisense RNA [17]. Additionally, fusion tags have been widely used to reduce protein toxicity, also increasing protein solubility [18,19]. Another approach that can be used for these difficult-to-produce or toxic proteins is the cell-free or in vitro recombinant protein synthesis system, which has been extensively described by Smolskaya et al. [20]. The *E. coli* extract-based cell-free expression system is the most popular, and it has significantly improved during the last few years. Two main advantages of this system are that there is no need to support cellular metabolism and that the reaction environment can be controlled, making it possible to add any necessary component into the reaction mixture to reach the optima protein production conditions.

The aggregation of recombinant proteins in the form of inclusion bodies (IBs) is also a major issue associated with recombinant production processes. Some proteins show a low solubility and are mainly produced in their aggregated version. In consequence, many solubilization strategies and a wide number of refolding parameters have been described for the isolation of soluble proteins from IBs [21]. Due to the extent of IB characterization in the last two decades, showing that IBs are protein aggregates formed (at least partially) by correctly folded and fully active recombinant proteins [22], non-denaturing solubilization protocols have been recently developed [23,24].

Finally, emerging tools are also being used to optimize the expression of recombinant proteins. The use of high-throughput technologies allows the reshaping and optimization of traditional production processes in a fast and efficient way. This includes cultivation platforms, detection methods, and screening systems [25]. In addition, the use of synthetic biology to design and build genome-reduced bacteria is an interesting technology to design bacteria with a minimal genome removing the unnecessary proportions of genome which can improve cellular capacity, and, consequently, increase heterologous protein expression [26].
